# Somatic and germline mutations in endometrial cancer

**DOI:** 10.25122/jml-2024-0313

**Published:** 2024-06

**Authors:** Robert Botea, Madalina Piron-Dumitrascu, Tiberiu Augustin Georgescu, Camil Laurentiu Bohiltea, Silviu Cristian Voinea, Valentin Nicolae Varlas, Simona Raluca Iacoban, Nicolae Suciu

**Affiliations:** 1 Department of Obstetrics and Gynecology, Carol Davila University of Medicine and Pharmacy, Bucharest, Romania; 2 Department of Obstetrics and Gynecology, Alessandrescu-Rusescu National Institute of Mother and Child Health, Bucharest, Romania; 3 Department of Pathology, Carol Davila University of Medicine and Pharmacy, Bucharest, Romania; 4 Department of Pathology, Alessandrescu-Rusescu National Institute of Mother and Child Health, Bucharest, Romania; 5 Department of Medical Genetics, Carol Davila University of Medicine and Pharmacy, Bucharest, Romania; 6 Materno-Fetal Assistance Excellence Center, Alessandrescu-Rusescu National Institute of Mother and Child Health, Bucharest, Romania; 7 Department of General Surgery, Carol Davila University of Medicine and Pharmacy, Bucharest, Romania; 8 Department of Oncological Surgery, Alexandru Trestioreanu Oncology Institute, Bucharest, Romania; 9 Department of Obstetrics and Gynecology - Filantropia Obstetrics and Gynecology Clinical Hospital, Carol Davila University of Medicine and Pharmacy, Bucharest, Romania

**Keywords:** Endometrial cancer, whole exome sequencing, germline mutations, somatic mutations, cancer genetics, personalized medicine

## Abstract

Endometrial cancer is a complex disease influenced by both somatic and germline mutations. While individual mutations in genes such as *PTEN, PIK3CA*, and members of the DNA mismatch repair (MMR) system have been extensively studied, comprehensive analyses comparing somatic and germline mutations within the same cohort are limited. This study compares these mutations using whole exome sequencing (WES) data from tumor and blood samples in patients with endometrial cancer. Thirteen female patients with histologically confirmed endometrial cancer were selected. Tumor tissues and matched blood samples were collected and subjected to WES at the CeGaT laboratory, followed by bioinformatics analysis and annotation using the Geneyx platform. WES revealed significant somatic and germline DNA mutations, with key pathogenic variants identified in genes such as *PTEN, PIK3CA, TP53, MLH1*, and *MSH2*. Comparative analysis showed distinct and overlapping mutation profiles, highlighting the importance of integrating somatic and germline data in endometrial cancer research.

## INTRODUCTION

Endometrial cancer is the most common gynecological malignancy in developed countries, with an estimated 65,620 new cases and 12,590 deaths in the United States in 2020 alone [1]. The incidence of endometrial cancer has been rising, partly due to the increased prevalence of risk factors such as obesity, hypertension, and diabetes [2]. While the majority of cases are diagnosed at an early stage and have a favorable prognosis, a significant proportion presents with advanced disease, which is associated with poor outcomes [3,4].

Genetic factors play a crucial role in the development of endometrial cancer [5,6]. These factors can be broadly categorized into germline and somatic mutations. Germline mutations are inherited and present in every cell of the body, whereas somatic mutations are acquired and confined to the tumor cells [7,8]. Distinguishing between these two types of mutations is essential for understanding the genetic basis of endometrial cancer and developing targeted therapies and personalized treatment plans [9].

Recent advances in genomic technologies have facilitated the identification of numerous somatic and germline mutations associated with endometrial cancer. Somatic mutations commonly found in endometrial cancer include phosphatase and tensin homolog *PTEN, PIK3CA, ARID1A*, and *TP53* alterations. These mutations play critical roles in tumorigenesis, affecting key pathways such as the PI3K/AKT and p53 signaling pathways [10-12].

On the germline front, mutations in mismatch repair (MMR) genes, such as *MLH1, MSH2, MSH6*, and *PMS2*, are well-established causes of Lynch syndrome, significantly increasing the risk of endometrial cancer. Women with Lynch syndrome have a lifetime risk of endometrial cancer that ranges from 40% to 60% [13]. Also, germline mutations in genes like *BRCA1, BRCA2*, and *PTEN* (associated with Cowden syndrome) contribute to hereditary endometrial cancer risk [14].

Whole exome sequencing (WES) has emerged as a powerful tool for identifying genetic mutations. By sequencing the protein-coding regions of the genome, WES can uncover both common and rare mutations, providing a comprehensive view of the genetic alterations involved in cancer [15]. This technology has been instrumental in identifying novel cancer-related genes and understanding the complex genetic landscape of various malignancies, including endometrial cancer [16].

Despite the advancements in identifying somatic and germline mutations separately, there is a notable lack of comprehensive studies that compare these mutations within the same cohort of patients with endometrial cancer. Most studies focus on either somatic or germline mutations, which limits our understanding of how these genetic alterations interact and contribute to cancer development and progression. A detailed comparison of somatic and germline mutations within the same patients would provide valuable insights into the genetic etiology of endometrial cancer and help identify potential targets for therapy. This study aims to identify and compare somatic and germline mutations in endometrial cancer using whole exome sequencing data from both tumor and blood samples. By analyzing WES data from 13 patients with endometrial cancer, this study aims to elucidate the genetic differences and interactions between somatic and germline mutations, thereby advancing our understanding of the genetic basis of endometrial cancer and informing the development of personalized treatment strategies.

## MATERIAL AND METHODS

### Patient selection

Thirteen female patients with histologically confirmed endometrial cancer were selected for this study. The inclusion criteria were as follows:

1. Patients must have a confirmed diagnosis of endometrial cancer based on histopathological examination.
2. Patients should not have received any prior treatment (surgery, chemotherapy, or radiation) for their endometrial cancer to ensure that the genetic analysis reflects the untreated tumor profile.
3. Patients with a strong family history of Lynch syndrome-associated cancers (e.g., colorectal, ovarian, gastric) were prioritized to enrich the study population with potential germline mutations.
4. Patients consented to participate in genetic studies and the use of their samples for research purposes.

Exclusion criteria

1. Previous cancer treatments: patients who have had chemotherapy, radiotherapy, or systemic cancer treatment before sample collection to avoid additional genetic mutations.
2. Metastatic disease: patients with metastatic endometrial cancer at diagnosis, as it can alter the primary tumor's genetic profile.
3. Inadequate sample quality: patients whose tumor or blood samples do not meet DNA integrity and purity standards.
4. Incomplete clinical data: lacking comprehensive clinical data, including family history and treatment records.

### Ethical approval and approval details

The study protocol was approved by the Institutional Review Board (IRB) of the Alessandrescu-Rusescu National Institute of Mother and Child Health, Bucharest, Romania, ensuring compliance with ethical standards for human research. All patients provided written informed consent before participating in the study, including consent for genetic testing and publication of anonymized data. The study was conducted in accordance with the Declaration of Helsinki and Good Clinical Practice guidelines.

### Sample collection

#### Blood and tumor tissue collection

Peripheral blood samples (5 ml) were collected from each patient using EDTA tubes to prevent coagulation. Tumor tissue samples were obtained through biopsy or surgical resection, ensuring samples were collected under sterile conditions to prevent contamination. All samples were immediately stored at 4°C until DNA extraction.

### Whole exome sequencing (WES)

#### DNA extraction process

Thin sections of formalin-fixed, paraffin-embedded (FFPE) tumor tissues and matched blood samples were collected and shipped to CeGaT GmbH in Tübingen, Germany. The DNA extraction from FFPE tissues was performed using the MagMax FFPE DNA/RNA Ultra (Thermo Fisher). The DNA extraction from blood on EDTA was performed using the QIA Symphony DSP DNA Mini Kit 96, Version 1 (Qiagen), following the manufacturer's protocol. The extracted DNA was quantified using a fluorescence-based quantification method. All samples passed quality control and proceeded to library preparation, where 50 ng of DNA was used for each sample. The libraries were prepared using the CeGaT Exome V5 kit from Twist Bioscience.

### WES protocol

Sequencing was conducted on the Illumina NovaSeq 6000 platform with paired-end reads of 101 base pairs each, resulting in high-quality data with a Q30 value exceeding 89.78%. To ensure the accuracy of base calling, particularly at the ends of the reads, an additional base was sequenced in both reads 1 and 2, leading to a configuration of 2 x 101 bp rather than the standard 2 x 100 bp. This approach was adopted to enhance the overall quality score calculation for the final base in each read, thus ensuring the reliability of the sequencing data for subsequent analysis. Demultiplexing of the sequencing reads was performed using Illumina's bcl2fastq software (version 2.20). In cases where more sequencing output was generated than initially requested, the reads for those samples were downsampled to ensure that the output remained at least 20% above the ordered quantity. Adapter sequences were trimmed using Skewer (version 0.2.2). Subsequent quality control of the FASTQ files was conducted on Illumina’s DRAGEN Bio-IT Platform (version 4.2.4). The quality of the sequencing data was rigorously assessed using FastQC, which provided detailed metrics on read quality, GC content, and sequence duplication levels.

### Bioinformatics analysis

Whole exome sequencing data as raw FASTQ files were processed using the Geneyx Analysis platform (Geneyx Genomex), version 5.17, integrated with the DRAGEN (Dynamic Read Analysis for GENomics) pipeline (Illumina), version 3.7.5. The FASTQ data was aligned to the human reference genome (hg19/GRCh37) using DRAGEN, which provides high-speed and accurate read mapping, variant calling, and quality control. Variant detection included single nucleotide variants (SNVs), insertions, and deletions. The resulting variant call format (VCF) files were subjected to further annotation and interpretation within Geneyx, utilizing databases such as ClinVar, dbSNP, and OMIM. Predictive tools, including PolyPhen-2, SIFT, and CADD, were employed to assess variant pathogenicity. Variants were classified according to American College of Medical Genetics and Genomics (ACMG) guidelines, focusing on those relevant to endometrial cancer.

### Variant annotation and interpretation

Variants were classified based on the ACMG guidelines [17]. Only variants categorized as pathogenic, likely pathogenic, or variants of uncertain significance were included in the final analysis.

An array of databases and bioinformatics tools were employed to facilitate the analysis. ClinVar was also utilized for clinical significance annotations, especially regarding the pathogenicity of genetic variants. To incorporate known variant information, dbSNP was used. Functional impact predictions were carried out using PolyPhen-2, SIFT, and CADD. The Online Mendelian Inheritance in Man (OMIM) database was also consulted to gather information on gene-disease associations.

### Statistical analysis

Descriptive statistics were used to summarize patient demographics and variant distributions. Fisher’s exact test was applied to compare the frequency of variants between germline and somatic samples. A *P* value of less than 0.05 was considered statistically significant. Variants with high pathogenicity scores and significant clinical correlations were prioritized for further functional validation. The results were interpreted in the context of their potential impact on endometrial cancer pathogenesis and their relevance for clinical management and genetic counseling..

## RESULTS

### Patient demographics and clinical data

The study cohort comprised 13 female patients with histologically confirmed endometrial cancer. The median age at diagnosis was 57 years, ranging from 47 to 75 years. All patients were of European descent, with diverse backgrounds representing different regions.

All of the patients presented with endometrioid carcinoma, the most common histological subtype of endometrial cancer.

### Overview of germline and somatic variants

The whole exome sequencing analysis of the 13 patients revealed a total of 731 variants (329 germline variants and 402 somatic variants) across the cohort. Germline variants were identified in all patients, with a median of 24 variants per patient (IQR: 18-42). These included known pathogenic variants as well as variants of uncertain significance. Somatic variants were more numerous, with a median of 31 variants per patient (IQR: 17-43), reflecting the genomic instability characteristic of tumor cells.

**Figure 1:**
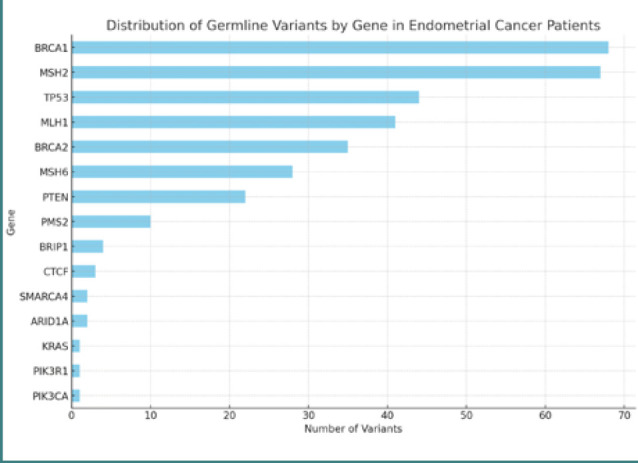
Distribution of germline variants by gene in patients with endometrial cancer

Most germline variants were found in genes associated with DNA repair mechanisms, such as M *LH1, MSH2, MSH6, PMS2*, and *BRCA1/2* (Figure 1).

**Figure 2:**
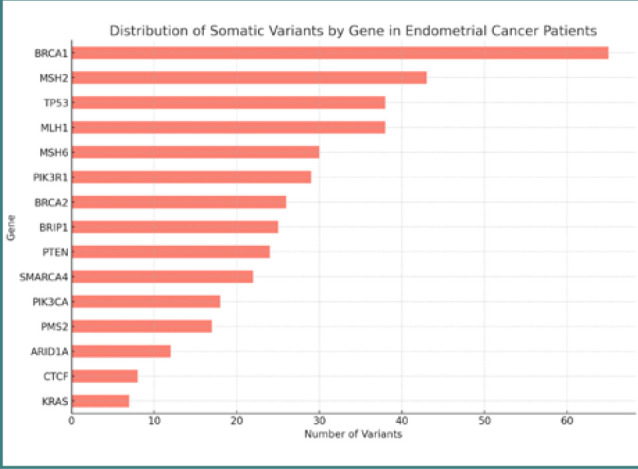
Distribution of somatic variants by gene in patients with endometrial cancer

In contrast, somatic samples included more variants in genes involved in cell cycle regulation, signal transduction pathways, and chromatin remodeling, including *TP53, PTEN, PIK3CA, ARID1A*, and *KRAS* (Figure 2).

Table 1 summarizes the 20 most frequent germline variants in patients with endometrial cancer. The table includes the gene in which each variant is located, the specific variant (HGVSC), the number of patients exhibiting the variant, and the frequency percentage of each variant within the patient population. The variants are ordered by frequency, with the highest frequency variants listed first.

**Table 1:** Distribution of the top 20 germline variants by frequency in patients with endometrial cancer

Gene	Variant	Number of Patients	Frequency %
*TP53*	c.^*^772del	13	100
*TP53*	c.376-160_376-158del	13	100
*TP53*	c.993+408_993+409dup	13	100
*PMS2*	c.706-4del	9	69.23
*MSH6*	c.3557-4dup	9	69.23
*BRCA1*	c.4987-196dup	9	69.23
*MSH6*	c.4002-10del	9	69.23
*MLH1*	c.1039-33_1039-26del	9	69.23
*MLH1*	c.1409+1224_1409+1227del	9	69.23
*MLH1*	c.678-241_678-234dup	8	61.54
*BRCA2*	c.7007+2436dup	7	53.85
*PTEN*	c.^*^1458_^*^1459del	6	46.15
*MSH2*	c.1277-3287_1277-3286del	6	46.15
*BRCA1*	c.441+64del	6	46.15
*BRCA1*	c.81-3498del	6	46.15
*BRCA1*	c.441+36_441+49del	5	38.46
*MSH2*	c.1276+132del	5	38.46
*BRCA1*	c.^*^872_^*^873del	5	38.46
*MSH2*	c.942+26_942+29del	5	38.46
*BRCA1*	c.671-248_671-246dup	5	38.46

Table 2 presents the 20 most frequent somatic variants identified in patients with endometrial cancer. The table lists the gene in which the variant is present, the specific variant (HGVSC), the number of patients carrying the variant, and the frequency percentage of each variant within the patient cohort. Variants are sorted in descending order of frequency, highlighting the most prevalent mutations in this population.

**Table 2:** Distribution of the top 20 somatic variants by frequency in patients with endometrial cancer

Gene	Variant	Number of Patients	Frequency %
*TP53*	c.993+408_993+409dup	9	69.23
*MLH1*	c.1409+1224_1409+1227del	9	69.23
*BRCA1*	c.81-3498del	8	61.54
*PIK3R1*	c.335-21006dup	8	61.54
*TP53*	c.376-160_376-158del	8	61.54
*BRCA1*	c.441+64del	7	53.85
*PIK3R1*	c.917-3329A>G	7	53.85
*MSH6*	c.4002-10del	6	46.15
*BRCA1*	c.^*^872_^*^873del	6	46.15
*BRIP1*	c.^*^2815_^*^2816insAAGAAA	6	46.15
*TP53*	c.^*^772del	6	46.15
*BRCA1*	c.441+36_441+49del	6	46.15
*PMS2*	c.706-5_706-4del	5	38.46
*TP53*	c.376-161_376-158del	5	38.46
*MSH6*	c.3557-4dup	5	38.46
*BRIP1*	c.1140+91dup	5	38.46
*PMS2*	c.706-5del	5	38.46
*MSH2*	c.1276+132del	5	38.46
*PIK3R1*	c.503-93_503-92del	5	38.46
*PIK3CA*	c.1540-55C>T	5	38.46

### Comparison of the number and types of variants found in germline vs. somatic samples

In this study, we analyzed germline variants of uncertain significance in patients with endometrial cancer to elucidate potential genetic contributors to this malignancy. The top 20 variants were identified based on frequency across the patient cohort. Notably, the *BRCA1* gene emerged as the most frequently mutated gene in this category, with 68 instances of the c.4987-196dup variant, accounting for 69.23% of the patient population. This was followed closely by mutations in *MSH2* and *TP53* found in 67 and 44 patients, respectively.

Among other findings, the *MLH1* c.1321G>A (p.Ala441Thr) variant was found in one patient and is probable to impair the ATPase activity of *MLH1*, which is crucial for its role in mismatch repair. The *MSH2* c.1077-10T>C variant, with a CADD Phred score of 22.4, was detected in a patient and is predicted to cause aberrant splicing, leading to a loss of function. Additionally, the *BRCA2* c.10095_10096insT (p.Ser3366fs*1) variant was found in a patient, and the frameshift is predicted to have a high impact on the protein.

Our analysis of somatic variants in patients with endometrial cancer revealed several key mutations that could have significant implications for understanding the disease and guiding treatment. Among the top 20 most frequent somatic variants, the *TP53* gene was identified as the most commonly mutated, with the c.993+408_993+409dup variant present in 69.23% of the patient cohort. This finding aligns with the established role of *TP53* as a tumor suppressor and its frequent mutation in various cancers, including endometrial cancer.

Additionally, mutations in the *MLH1* gene, specifically the c.1409+1224_1409+1227del variant, were also prevalent, appearing in 69.23% of patients. The high frequency of *MLH1* mutations is particularly noteworthy given their association with microsatellite instability (MSI) and Lynch syndrome, which are known to contribute to endometrial carcinogenesis. Identifying *PIK3R1* mutations in 61.54% of patients further emphasizes the role of the PI3K/AKT/mTOR pathway in this cancer type, supporting the rationale for ongoing research into targeted therapies that inhibit this pathway.

### Comparative analysis

Regarding the number of variants, somatic samples exhibited a higher average number of variants compared to germline samples, reflecting the increased genomic instability and mutation rates in tumor cells. The median number of somatic variants was 31 per patient (IQR: 17-43), significantly higher than the median of 24 germline variants per patient (IQR: 18-42). The mean difference between the two groups was 5.6 variants (95% CI, 4.8–6.4). This difference was statistically significant (*P* = 0.03, Mann-Whitney U test), highlighting the elevated mutational burden in somatic cells.

The comparative analysis of germline and somatic variants revealed a diverse range of mutation types, with notable differences in their frequencies. Deletions (del) were the most common mutation type in germline and somatic samples, comprising 42.25% and 41.29% of the variants, respectively. Duplications (dup) were more prevalent in germline samples, accounting for 27.96% of the variants, compared to 18.91% in somatic samples. Single nucleotide polymorphisms (SNPs) were significantly more frequent in somatic samples, representing 37.81% of the total variants, while they constituted 28.88% in germline DNA. Insertions (ins) were relatively rare in both groups but were slightly more frequent in somatic samples at 1.99%, compared to 0.91% in germline samples (Table 3).

**Table 3:** Distribution of germline and somatic variants by type in endometrial cancer cohort

Variant Type	Germline	%	Somatic	%	Total
Deletion	139	42.25%	166	41.29%	305
Duplication	92	27.96%	76	18.91%	168
Insertion	3	0.91%	8	1.99%	11
Single Nucleotide Variant	95	28.88%	152	37.81%	247
Total	329		402		731

The germline and somatic variants analysis revealed distinct mutation patterns across different mutation types. Intronic variants were the most prevalent in germline and somatic samples, constituting 76.9% and 64.93% of the total variants, respectively. A notable difference was observed in the frequency of frameshift mutations, which were entirely absent in germline samples but accounted for 2.74% of the somatic variants. Similarly, non-synonymous coding variants were more common in somatic samples, representing 4.48% of the variants, compared to just 0.61% in germline DNA. Additionally, stop-gained mutations were present exclusively in somatic samples at a frequency of 1.24%, while a single frame shift-stop-gained mutation was detected in the germline at a frequency of 0.30%.

Variants affecting splice site regions were also more frequently observed in somatic samples, with 8.96% of variants falling into this category, compared to 6.08% in germline samples. UTR (untranslated region) variants were fairly consistent between the two groups, with UTR 3' prime variants comprising 10.64% of germline and 9.70% of somatic variants. Interestingly, start-gained mutations were identified in somatic samples (0.50%) but were absent in germline DNA. These findings highlight the differential mutation landscapes between germline and somatic genomes in endometrial cancer, emphasizing the importance of somatic mutations in the tumorigenic process.

Pathway analysis revealed distinct patterns of involvement in germline and somatic variants. Germline variants were primarily associated with DNA repair pathways, highlighting the inherited predisposition to cancer through compromised genomic maintenance mechanisms. Somatic variants, on the other hand, were enriched in pathways related to cell cycle regulation, signal transduction, and chromatin remodeling, indicating the acquired mutations that drive tumor progression and metastasis.

### Pathogenic variants

The analysis of WES data from the 13 patients with endometrial cancer revealed several key pathogenic variants in the somatic samples. These variants were identified in genes known to play critical roles in tumorigenesis and cancer progression.

The analysis of somatic pathogenic variants identified several key mutations in genes commonly associated with endometrial cancer. Among the detected variants, the *PIK3CA* gene harbored the c.3140A>G mutation, resulting in the p.His1047Arg amino acid change, which is classified as likely pathogenic. This specific variant is known for its role in activating the PI3K/AKT signaling pathway, contributing to tumorigenesis.

*KRAS* mutations were also prevalent, with two notable variants: c.35G>T (p.Gly12Val), categorized as likely pathogenic, and c.38G>A (p.Gly13Asp), which is classified as pathogenic. These mutations are critical drivers of oncogenesis, particularly through the MAPK/ERK signaling pathway, influencing cell proliferation and survival.

*PTEN*, a tumor suppressor gene frequently mutated in endometrial cancer, showed multiple pathogenic variants, including c.376G>T (p.Ala126Ser) and c.389G>A (p.Arg130Gln), both classified as likely pathogenic and c.203A>G (p.Tyr68Cys), which is classified as pathogenic. These mutations are significant as they result in the loss of *PTEN’s* tumor-suppressive function, leading to uncontrolled cellular growth.

Lastly, a mutation in *TP53*, another critical tumor suppressor gene, was identified as c.733G>A, leading to the p.Gly245Ser change, classified as likely pathogenic. Mutations in *TP53* are associated with a wide range of cancers and are indicative of poor prognosis due to their role in disrupting DNA repair and apoptosis mechanisms.

These findings underscore the complexity and heterogeneity of somatic mutations in endometrial cancer, with a combination of both likely pathogenic and pathogenic variants in genes crucial to cancer development and progression.

### Novel variants

The WES analysis identified several novel variants not previously associated with endometrial cancer. These novel variants, found in both germline and somatic samples, potentially contribute to the pathogenesis of the disease.

Among the findings, we identified a germline variant, previously unreported in dbSNP, in the *MSH2* gene (c.2005+61dup) found in one patient. This variant is intronic and is predicted to have a low-severity impact on the protein.

Additionally, in this study, several novel somatic variants were identified in key genes associated with endometrial cancer, many of which have not been previously reported or classified in major databases like ClinVar or dbSNP. These variants were predominantly frameshift deletions and nonsense mutations, leading to significant alterations in protein function, all classified with high severity.

*ARID1A*, a tumor suppressor gene frequently mutated in various cancers, exhibited multiple novel variants. Among these, the c.3189_3190delinsC variant was identified, leading to a p.Leu1064fs frameshift and classified as a variant of uncertain significance (VUS). Similarly, the c.3745_3757del (p.Gly1249fs16) and c.5548del (p.Asp1850fs33) deletions were detected, both causing frameshift mutations. Another notable *ARID1A* variant, c.5320G>T (p.Glu1774*), resulted in a premature stop codon and was also classified as VUS, but its impact as a stop-gained mutation suggests potential loss of function. These findings highlight the recurrent involvement of *ARID1A* in endometrial carcinogenesis, with these novel variants potentially contributing to tumor progression through disruption of chromatin remodeling.

*BRIP1*, another gene implicated in DNA repair, was found to harbor the c.104_108del (p.Gly35fs*32) variant, leading to a frameshift and premature truncation of the protein. This novel variant was classified as VUS, but given the role of *BRIP1* in maintaining genomic stability, its high-severity classification suggests a likely impact on protein function.

*MLH1*, a critical gene in the DNA mismatch repair pathway, also showed novel variants, including the c.2113_2141del (p.Pro705fs*8) frameshift mutation and the c.514G>A (p.Glu172Lys) missense mutation, the latter being classified as likely pathogenic by ClinVar. These mutations are of particular interest given *MLH1*'s role in maintaining genomic integrity, and their identification in this study underscores the potential for novel disruptions in mismatch repair contributing to tumorigenesis.

*PIK3R1*, involved in the PI3K/AKT signaling pathway, exhibited the c.1669C>T (p.Arg557*) stop-gained mutation and the c.1732_1733del (p.Asp578fs*23) frameshift deletion. Both variants are classified as VUS, but their predicted impact suggests significant functional impairment, potentially affecting pathway signaling and contributing to cancer cell survival and proliferation.

Finally, *PTEN*, another well-known tumor suppressor gene, was found to have a novel frameshift deletion, c.923_936del (p.Arg308fs*12), classified as VUS. Given *PTEN*'s role in negatively regulating the PI3K/AKT pathway, this mutation could have profound effects on cellular growth and apoptosis regulation, further implicating *PTEN* loss as a driver in endometrial cancer.

These findings reveal a spectrum of novel somatic variants in genes critical to cancer development, many of which carry high-severity predictions. While classified as variants of uncertain significance, the potential impact of these mutations on protein function and their involvement in key cancer-related pathways suggest they may play important roles in the pathogenesis of endometrial cancer. Further functional studies will be necessary to elucidate their exact contribution to tumorigenesis.

## DISCUSSION

This comprehensive analysis of whole exome sequencing data from 13 patients with endometrial cancer reveals significant insights into the genetic landscape of the disease, highlighting both somatic and germline mutations and their implications for tumor development and progression.

This study provides a comprehensive comparison of somatic and germline mutations in endometrial cancer using WES data from both tumor and blood samples. A total of 731 variants were identified across 13 patients, with 329 germline and 402 somatic variants, reflecting the distinct mutation landscapes between inherited and acquired genetic alterations in endometrial cancer. A key finding of this study is the significantly higher number of somatic variants compared to germline variants, with a median of 31 somatic mutations per patient compared to 24 germline mutations. This difference underscores the genomic instability characteristic of tumor cells, which leads to a higher mutational burden and contributes to cancer progression. The statistical significance of this difference (*P* = 0.03) highlights the importance of considering somatic mutations in the context of endometrial cancer's pathogenesis. The mutation types also varied between germline and somatic samples. Deletions were the most common mutation type in both groups; however, single nucleotide polymorphisms were significantly more frequent in somatic samples, suggesting that point mutations play a more prominent role in tumorigenesis. Conversely, duplications were more prevalent in germline samples, indicating that these may be more related to inherited cancer risk.

Furthermore, the study identified distinct pathways affected by germline and somatic mutations. Germline mutations were predominantly found in DNA repair genes, consistent with the role of inherited mutations in predisposition to endometrial cancer through compromised genomic maintenance. In contrast, somatic mutations were enriched in genes involved in cell cycle regulation, signal transduction, and chromatin remodeling, highlighting their role in the acquired traits that drive tumor progression.

Several key pathogenic variants were identified in somatic samples, including mutations in well-known cancer-associated genes such as *PIK3CA, KRAS, PTEN*, and *TP53*. These mutations are critical drivers of oncogenesis, influencing pathways like PI3K/AKT and MAPK/ERK, which are integral to cell proliferation, survival, and tumor growth. Importantly, the study also identified novel somatic variants in genes like *ARID1A, BRIP1, MLH1, PIK3R1*, and *PTEN*, many of which have not been previously reported or classified. Although these novel variants are currently of uncertain significance, their high severity predictions and involvement in key cancer-related pathways suggest that they may contribute significantly to the pathogenesis of endometrial cancer.

These findings collectively advance our understanding of the genetic landscape of endometrial cancer, illustrating the distinct and overlapping roles of germline and somatic mutations in the disease. The identification of novel variants further underscores the complexity of the genetic alterations involved and highlights the need for continued research to fully elucidate their roles in cancer development and progression. There are significant clinical implications for the diagnosis, management, and treatment of endometrial cancer, particularly in personalized medicine. The identification of both germline and somatic mutations provides a comprehensive understanding of the genetic underpinnings of the disease, which is crucial for developing targeted therapies and improving patient outcomes.

The presence of germline mutations in DNA repair genes, such as *MLH1, MSH2*, and *BRCA2*, underscores the importance of genetic testing for hereditary cancer syndromes like Lynch syndrome and Cowden syndrome in patients diagnosed with endometrial cancer. Identifying these germline mutations can help stratify patients based on their genetic risk, enabling early detection and preventative measures for patients and their at-risk family members. Moreover, the detection of novel germline variants, such as the previously unreported *MSH2* c.2005+61dup, highlights the need for ongoing research and updates to genetic screening panels to incorporate emerging variants that may influence cancer risk.

The discovery of somatic mutations in key oncogenes and tumor suppressor genes, including *PIK3CA, KRAS, PTEN*, and *TP53*, has direct implications for tumor profiling and the development of personalized treatment strategies. For example, the *PIK3CA* c.3140A>G (p.His1047Arg) mutation is known to activate the PI3K/AKT pathway, which is a critical driver of oncogenesis in endometrial cancer. Targeting this pathway with PI3K inhibitors, such as alpelisib, which has shown efficacy in other cancers, represents a promising therapeutic approach for patients harboring this mutation [18].

Similarly, *KRAS* mutations, particularly the pathogenic c.35G>T (p.Gly12Val) variant identified in this study, are well-known drivers of cancer and are associated with resistance to certain therapies, such as anti-EGFR monoclonal antibodies. However, emerging therapeutic options targeting *KRAS*, including *KRAS* G12C inhibitors, offer new hope for patients with these mutations, and ongoing clinical trials may soon provide new standard-of-care options [19].

The identification of novel somatic variants, particularly in genes like *ARID1A* and *MLH1*, which play critical roles in chromatin remodeling and DNA mismatch repair, respectively, further emphasizes the need for personalized approaches in cancer treatment. Although these novel variants are currently classified as variants of uncertain significance, their potential impact on protein function suggests that they could be important biomarkers for predicting treatment response or resistance. For example, loss of function in *ARID1A*, as seen with the c.5320G>T (p.Glu1774*) variant, has been associated with sensitivity to EZH2 inhibitors, providing a potential therapeutic avenue that could be explored in future clinical trials [20].

The integration of germline and somatic mutation data into clinical decision-making processes is essential for advancing personalized medicine in endometrial cancer. The ability to tailor treatment strategies based on a patient's unique genetic profile enhances the effectiveness of therapies and minimizes the risk of adverse effects. For instance, patients with germline mutations in mismatch repair genes may benefit from immunotherapy with PD-1 inhibitors, such as pembrolizumab, which has been shown to be effective in tumors with high microsatellite instability (MSI) [21]. Furthermore, identifying novel somatic variants that are not yet well-characterized presents an opportunity for further research and the potential development of new targeted therapies. These findings suggest that endometrial cancer may be driven by a wider array of genetic alterations than previously understood, and expanding the scope of genetic testing could lead to the discovery of additional actionable mutations.

The trend towards comprehensive genomic profiling in cancer care will likely continue, with whole exome sequencing playing a central role in identifying known and novel mutations. The use of WES to simultaneously analyze germline and somatic mutations within the same cohort, as demonstrated in this study, represents a significant advancement in understanding the full spectrum of genetic alterations involved in endometrial cancer. This approach not only provides a more complete picture of the disease but also opens the door to new therapeutic possibilities that could transform patient care and highlights the critical importance of integrating genomic data into the clinical management of endometrial cancer. By identifying key mutations and novel variants, this research paves the way for more personalized and effective treatment strategies, ultimately improving outcomes for patients with this challenging disease.

The findings from this study align with and expand upon the current understanding of the genetic landscape of endometrial cancer as reported in the literature. The identification of somatic mutations in key oncogenes and tumor suppressor genes such as *PIK3CA, KRAS, PTEN*, and *TP53* is consistent with previous studies that have highlighted the central role of these mutations in endometrial cancer pathogenesis [10,11]. For example, the *PIK3CA* mutation c.3140A>G (p.His1047Arg) identified in this study is one of the most frequently reported mutations in endometrial cancer and has been extensively documented as a driver of the PI3K/AKT pathway, contributing to tumorigenesis and cancer progression [11]. Similarly, *KRAS* mutations, particularly the c.35G>T (p.Gly12Val) variant, have been recognized in a variety of cancers, including endometrial cancer, where they play a significant role in oncogenic signaling [11].

The *PTEN* and *TP53* mutations identified in this study also corroborate findings from previous research. *PTEN* mutations, such as the c.203A>G (p.Tyr68Cys) variant identified here, are known to result in the loss of tumor-suppressive functions, thereby facilitating uncontrolled cell proliferation. This finding is consistent with the established role of *PTEN* in endometrial carcinogenesis, where it is frequently mutated, particularly in endometrioid subtypes of the disease [22,23]. The *TP53* mutation c.733G>A (p.Gly245Ser), which disrupts the DNA-binding domain of the *TP53* protein, aligns with existing literature that links *TP53* mutations to poor prognosis and resistance to therapy in various cancers, including endometrial cancer [24].

The identification of novel variants in this study adds a new dimension to the existing body of knowledge. The *ARID1A* variants identified, including the c.3189_3190delinsC (p.Leu1064fs) and c.5320G>T (p.Glu1774*) mutations, highlight the ongoing relevance of chromatin remodeling genes in endometrial cancer, a finding that has been previously reported but remains an area of active research. While *ARID1A* mutations are known to contribute to cancer through the disruption of the SWI/SNF chromatin remodeling complex, the novel variants identified in this study suggest that there may be additional, uncharacterized mechanisms by which *ARID1A* loss contributes to tumorigenesis [25].

The discovery of novel variants in *BRIP1, MLH1*, and *PIK3R1* further challenges the current understanding and suggests that endometrial cancer may involve a broader array of genetic alterations than previously recognized. For instance, the *MLH1* c.514G>A (p.Glu172Lys) variant classified as likely pathogenic in this study adds to the growing list of mutations associated with mismatch repair deficiency, a key feature in Lynch syndrome-associated cancers. This supports the notion that comprehensive genomic profiling could reveal additional mutations that may contribute to the cancer's etiology and progression, potentially leading to new biomarkers for early detection and novel therapeutic targets. However, some findings challenge existing assumptions, particularly in the context of variants of uncertain significance. The high frequency and severity of novel VUS, particularly in genes like *ARID1A* and *PTEN*, suggest that these variants may play a more critical role in endometrial cancer than currently understood. This underscores the need for further functional studies to validate the impact of these variants on protein function and cancer progression, as well as to reassess their classification as VUS. This study confirms much of what is known about the genetic drivers of endometrial cancer while also introducing novel findings that may challenge and expand the existing literature. The identification of both well-established and novel mutations underscores the genetic complexity of endometrial cancer and highlights the potential for future research to further elucidate the role of these variants in cancer development and patient outcomes.

This study makes several significant contributions to understanding the genetic basis of endometrial cancer, particularly by identifying novel variants that have not been previously associated with the disease. These novel variants, found in key genes involved in tumor suppression, DNA repair, and oncogenic signaling, provide new insights into the molecular mechanisms that may drive endometrial carcinogenesis.

Among the most noteworthy findings are the novel somatic variants identified in the *ARID1A* gene. *ARID1A*, a critical component of the SWI/SNF chromatin remodeling complex, has been frequently implicated in various cancers, including endometrial cancer [26,27]. The novel frameshift deletions and nonsense mutations identified in this study, such as c.3189_3190delinsC (p.Leu1064fs) and c.5320G>T (p.Glu1774*), introduce premature stop codons that likely result in the loss of *ARID1A* function. Given *ARID1A*’s role in maintaining chromatin structure and regulating gene expression, these mutations may disrupt normal cell cycle control and promote tumorigenesis. The identification of these variants contributes to the growing body of evidence that *ARID1A* loss is a critical event in the development of endometrial cancer, particularly in its more aggressive forms.

Similarly, the discovery of novel variants in *BRIP1* and *MLH1* underscores the importance of DNA repair mechanisms in maintaining genomic integrity and preventing cancer. The *BRIP1* c.104_108del (p.Gly35fs*32) variant, which results in a frameshift and subsequent loss of function, may compromise the cell’s ability to repair DNA double-strand breaks, leading to increased mutational burden and cancer risk. *MLH1*, a gene central to the mismatch repair pathway, exhibited the novel c.514G>A (p.Glu172Lys) variant, which was classified as likely pathogenic. This variant’s potential to impair *MLH1* function further supports its role in predisposing individuals to Lynch syndrome-associated cancers, including endometrial cancer. The identification of such novel variants in DNA repair genes adds to the understanding of how inherited and somatic mutations can synergize to drive cancer progression.

Another significant contribution of this study is the identification of novel variants in *PIK3R1* and *PTEN*, both of which are integral to the PI3K/AKT signaling pathway. The *PIK3R1* c.1669C>T (p.Arg557*) and *PTEN* c.923_936del (p.Arg308fs*12) mutations introduce premature stop codons, likely resulting in truncated proteins with diminished or absent function. Given the role of PI3K/AKT signaling in regulating cell growth, survival, and metabolism, these novel mutations may contribute to the dysregulation of this pathway, promoting uncontrolled cell proliferation and resistance to apoptosis. The identification of these variants highlights the potential for novel therapeutic targets within the PI3K/AKT pathway, which could be exploited in future treatment strategies for endometrial cancer. In addition to these findings, the study also identified a novel germline variant in *MSH2* (c.2005+61dup), which has not been previously reported in major databases like dbSNP. Although intronic and predicted to have a low severity impact, this variant may still play a role in the splicing regulation of *MSH2*, thereby contributing to the mismatch repair deficiency characteristic of Lynch syndrome. This discovery suggests that even variants outside the canonical coding regions can have significant implications for cancer risk and should be considered in comprehensive genetic screening.

Overall, the novel contributions of this study lie not only in the identification of previously unreported variants but also in the potential biological significance of these mutations. By expanding the catalog of genetic alterations associated with endometrial cancer, this research provides new avenues for exploring the molecular mechanisms underlying the disease. Furthermore, these findings may inform the development of novel diagnostic tools and targeted therapies, ultimately contributing to more personalized and effective treatment approaches for patients with endometrial cancer.

### Limitations

While this study offers valuable insights into the genetic landscape of endometrial cancer by comparing germline and somatic mutations, it has several limitations. The small sample size of 13 patients may not fully capture the genetic diversity of the disease, necessitating larger studies to validate these findings. Additionally, whole exome sequencing focuses only on coding regions, missing potential alterations in non-coding regions that could be critical to cancer development. The classification of several novel variants as variants of uncertain significance further highlights the need for functional validation to understand their impact on protein function and cancer progression. Furthermore, the exclusive focus on patients of European descent limits the applicability of the findings to more diverse populations, underscoring the need for studies involving ethnically diverse cohorts.

Future research should address these limitations by expanding the study to larger, more diverse patient cohorts and incorporating whole-genome sequencing (WGS) to explore non-coding regions. Functional validation of novel variants through in vitro and in vivo studies is essential to determine their role in cancer progression and potential as therapeutic targets. The findings underscore the importance of developing targeted therapies based on specific mutational profiles, particularly in the PI3K/AKT and MAPK/ERK pathways. By addressing these limitations, future studies can enhance our understanding of endometrial cancer's genetic basis and contribute to the development of more effective, personalized treatment strategies.

## CONCLUSION

This study provides a comprehensive analysis of the genetic alterations in endometrial cancer by examining both somatic and germline mutations through whole exome sequencing in a cohort of 13 patients. A total of 731 variants were identified, with 329 being germline and 402 somatic, illustrating a higher mutational burden in somatic samples, indicative of the genomic instability in tumor cells. The study underscores the critical roles of inherited and acquired mutations in the development and progression of endometrial cancer. Germline mutations predominantly impacted DNA repair pathways, emphasizing the importance of inherited predispositions in genes associated with Lynch syndrome and other hereditary cancer syndromes. On the other hand, somatic mutations were enriched in genes involved in cell cycle regulation, signal transduction, and chromatin remodeling, underlining their contribution to tumorigenesis and cancer progression.

The identification of novel variants in genes such as *ARID1A, BRIP1, MLH1, PIK3R1*, and *PTEN* expands the understanding of the genetic basis of endometrial cancer, suggesting that a broader spectrum of mutations may contribute to the disease than previously recognized. These findings have significant implications for clinical practice and personalized medicine. By analyzing both somatic and germline mutations, clinicians can better assess genetic risks and identify therapeutic vulnerabilities in individual patients. The identification of actionable mutations in pathways like PI3K/AKT and MAPK/ERK highlights the potential for targeted therapies tailored to the specific genetic profile of each patient. This dual focus on germline and somatic mutations enhances the understanding of endometrial cancer's genetic landscape and opens up new possibilities for more precise diagnostic tools, risk assessment strategies, and personalized treatment approaches that could improve patient outcomes.

## References

[1] Siegel RL, Miller KD, Jemal A (2020). Cancer statistics 2020. CA Cancer J Clin.

[2] Baker-Rand H, Kitson SJ (2024). Recent Advances in Endometrial Cancer Prevention, Early Diagnosis and Treatment. Cancers (Basel).

[3] Bokhman JV (1983). Two pathogenetic types of endometrial carcinoma. Gynecol Oncol.

[4] Lortet-Tieulent J, Ferlay J, Bray F, Jemal A (2018). International patterns and trends in endometrial cancer incidence, 1978-2013. J Natl Cancer Inst.

[5] Okuda T, Sekizawa A, Purwosunu Y, Nagatsuka M, Morioka M, Hayashi M, Okai T (2010). Genetics of endometrial cancers. Obstet Gynecol Int.

[6] Chang YS, Huang HD, Yeh KT, Chang JG (2016). Genetic alterations in endometrial cancer by targeted next-generation sequencing. Exp Mol Pathol.

[7] Briggs S, Tomlinson I (2013). Germline and somatic polymerase ε and δ mutations define a new class of hypermutated colorectal and endometrial cancers. J Pathol.

[8] Smith ES, Da Cruz Paula A, Cadoo KA, Abu-Rustum NR, Pei X, Brown DN (2019). Endometrial Cancers in BRCA1 or BRCA2 Germline Mutation Carriers: Assessment of Homologous Recombination DNA Repair Defects. JCO Precision Oncology.

[9] Morice P, Leary A, Creutzberg C, Abu-Rustum N, Darai E (2016). Endometrial cancer. Lancet.

[10] Le Gallo M, O'Hara AJ, Rudd ML, Urick ME, Hansen NF, O'Neil NJ (2012). Exome sequencing of serous endometrial tumors identifies recurrent somatic mutations in chromatin-remodeling and ubiquitin ligase complex genes. Nat Genet.

[11] Cancer Genome Atlas Research Network; Kandoth C, Schultz N, Cherniack AD (2013). Integrated genomic characterization of endometrial carcinoma. Nature.

[12] Markowska A, Pawałowska M, Lubin J, Markowska J (2014). Signalling pathways in endometrial cancer. Contemp Oncol (Pozn).

[13] Møller P, Seppälä T, Bernstein I (2017). Mallorca Group (http://mallorca-group.eu). Cancer incidence and survival in Lynch syndrome patients receiving colonoscopic and gynaecological surveillance: first report from the prospective Lynch syndrome database. Gut.

[14] Evans DG, Woodward ER, Bajalica-Lagercrantz S (2020). Germline TP53 Testing in Breast Cancers: Why, When and How?. Cancers (Basel).

[15] Wang Y, Du H, Dai W, Bao C, Zhang X, Hu Y (2023). Diagnostic Potential of Endometrial Cancer DNA from Pipelle, Pap-Brush, and Swab Sampling. Cancers (Basel).

[16] Wagle N, Berger MF, Davis MJ, Blumenstiel B, Defelice M (2012). High-throughput detection of actionable genomic alterations in clinical tumor samples by targeted, massively parallel sequencing. Cancer Discov.

[17] Richards S, Aziz N, Bale S, Bick D, Das S, Gastier-Foster J (2015). Standards and guidelines for the interpretation of sequence variants: a joint consensus recommendation of the American College of Medical Genetics and Genomics and the Association for Molecular Pathology. Genet Med.

[18] Chang DY, Ma WL, Lu YS (2021). Role of Alpelisib in the Treatment of PIK3CA-Mutated Breast Cancer: Patient Selection and Clinical Perspectives. Ther Clin Risk Manag.

[19] Ryan MB, Coker O, Sorokin A, Fella K, Barnes H, Wong E (2022). KRASG12C-independent feedback activation of wild-type RAS constrains KRASG12C inhibitor efficacy. Cell Rep.

[20] Bitler BG, Aird KM, Garipov A, Li H, Amatangelo M, Kossenkov AV (2015). Synthetic lethality by targeting EZH2 methyltransferase activity in ARID1A-mutated cancers. Nat Med.

[21] O'Malley DM, Bariani GM, Cassier PA, Marabelle A, Hansen AR, De Jesus Acosta A (2022). Pembrolizumab in Patients With Microsatellite Instability-High Advanced Endometrial Cancer: Results From the KEYNOTE-158 Study. J Clin Oncol.

[22] Gbelcová H, Gergely L, Šišovský V, Straka Ľ, Böhmer D, Pastoráková A (2022). PTEN mutations as predictive marker for the high-grade endometrial cancer development in slovak women. Physiol Res.

[23] Khatami F, Shahriari S, Aminimoghaddam S, Klashami ZN, Farahani MS, Teimoori-Toolabi L (2023). PTEN promoter methylation and expression in endometrial cancer tissues. Epigenomics.

[24] Mandilaras V, Garg S, Cabanero M, Tan Q, Pastrello C, Burnier J (2019). TP53 mutations in high grade serous ovarian cancer and impact on clinical outcomes: a comparison of next generation sequencing and bioinformatics analyses. Int J Gynecol Cancer.

[25] Jones S, Wang TL, Shih IeM, Mao TL, Nakayama K, Roden R (2010). Frequent mutations of chromatin remodeling gene ARID1A in ovarian clear cell carcinoma. Science.

[26] Toumpeki C, Liberis A, Tsirkas I, Tsirka T, Kalagasidou S, Inagamova L (2019). The Role of ARID1A in Endometrial Cancer and the Molecular Pathways Associated With Pathogenesis and Cancer Progression. In Vivo.

[27] Takeda T, Banno K, Okawa R, Yanokura M, Iijima M, Irie-Kunitomi H (2016). ARID1A gene mutation in ovarian and endometrial cancers (Review). Oncol Rep.

